# Multicenter Study of Antibiotic Resistance Profile of* H. pylori* and Distribution of CYP2C19 Gene Polymorphism in Rural Population of Chongqing, China

**DOI:** 10.1155/2016/8547686

**Published:** 2016-05-09

**Authors:** Ran Han, Hong Lu, Ming-Wan Jiang, Ke-Wen Tan, Zhong Peng, Jia-Li Hu, Dian-Chun Fang, Chun-Hui Lan, Xiao-Ling Wu

**Affiliations:** ^1^The Second Affiliated Hospital of Chongqing Medical University, Chongqing 400016, China; ^2^Department of Gastroenterology, Changshou District People's Hospital, Chongqing 400016, China; ^3^Department of Gastroenterology, Sanxia Central Hospital, Chongqing 400016, China; ^4^Department of Gastroenterology, Dianjiang County People's Hospital, Chongqing 400016, China; ^5^Department of Gastroenterology, Qijiang County People's Hospital, Chongqing 400016, China; ^6^Department of Gastroenterology, Southwest Hospital, Third Military Medical University, Chongqing 400016, China; ^7^Department of Gastroenterology, Daping Hospital, Third Military Medical University, Chongqing 400042, China

## Abstract

This study was to investigate the antibiotic resistance profile of* H. pylori* and the distribution of CYP2C19 gene polymorphism in rural population of Chongqing, China. 214 and 111 strains of* H. pylori* were isolated from rural and urban patients, respectively. 99.53%, 20.09%, and 23.36% of the isolates in rural patients were found to be resistant to metronidazole, clarithromycin, and levofloxacin, while the resistant rate in urban patients was 82.88%, 19.82%, and 24.32%. The multiple antibiotic resistance percentage significantly increased from 28.26% (below 45 years) to 41.80% (above 45 years) in rural patients. Up to 44.39%, 45.79%, and 9.81% of rural patients from whom* H. pylori* was isolated were found to be extensive metabolizers, intermediate metabolizers, and poor metabolizers. No correlation was observed between antibiotic resistance profile of* H. pylori* and genetic polymorphism of CYP2C19 among rural population. There was a high prevalence of* H. pylori* strains resistant to metronidazole, clarithromycin, and levofloxacin in rural patients in Chongqing, China. The choice of therapy in this area should be based on local susceptibility patterns. Amoxicillin, gentamicin, and furazolidone are recommended as the first-line empiric regimen.

## 1. Introduction

*Helicobacter pylori* is a gram-negative, helical, microaerophilic bacterium that colonizes the gastric mucosa of approximately 50% of the world's population [1]. The prevalence of* H. pylori* infection ranges from 25–50% in the developed countries to over 80% in the developing world. In China, an estimated 40 to 60% of population is infected by* H. pylori*, with an average infection rate of 54.76% [2]. Race, gender, age, geographical location, and socioeconomic status are known to be associated with the prevalence of* H. Pylori* infection. The organism has been implicated in the pathogenesis of gastric disorders such as chronic gastritis, peptic ulcer disease, gastric carcinoma, and gastric lymphoma [3, 4]. Epidemiological studies have demonstrated an association between* H. pylori* infection and an increased risk of gastric cancer [5]. According to a meta-analysis,* H. pylori* infection was found to be associated with a 2-fold increase in the risk for gastric cancer [6]. In a prospective study, approximately 2.9% of the patients infected with* H. pylori* eventually developed gastric cancer, while none of the patients without* H. pylori* infection developed gastric cancer [7]. At least 75% of new gastric cancer cases every year are thought to be linked to* H. pylori* infection [8]. It has been suggested that widespread treatment and elimination of* H. pylori* infection could protect against the occurrence of gastric cancer in people with no precancerous lesions [9, 10].

So far, triple therapy comprising a combination of two antibiotics (amoxicillin, clarithromycin, tinidazole, or metronidazole) and one proton pump inhibitor (PPI) (omeprazole, lansoprazole, pantoprazole, or rabeprazole) has yielded the best results in the treatment of* H. pylori* infection. However, with the recent emergence of antibiotic resistant strains of* H. pylori*, the efficacy of the standard triple therapy has reportedly decreased to <80% [11]. A retrospective analytic study revealed a progressive decrease in the* H. pylori* eradication rate achieved with the standard triple therapy (metronidazole, clarithromycin, and amoxicillin) in China, from 88.56% prior to year 2004, to 77.66% between 2005 and 2009, and further down to 71.13% from the year 2010 onwards [12]. Given the large population and the vast geographical expanse of China, the use of antibiotics for treatment of* H. pylori* infection is liable to vary greatly. Till date, the antibiotic resistance profile of* H. pylori* in the underdeveloped areas of China has not been documented. Thus, it is imperative to monitor antibiotic resistance of* H. pylori* in rural populations.

Proton pump inhibitors (PPIs) are known to be extensively metabolized in the liver by cytochrome P450 2C19 (CYP2C19). Although PPIs play an important role in improving the therapeutic efficacy of antibiotics for treatment of* H. pylori* infection, CYP2C19 gene polymorphism tends to affect both the pharmacokinetic and pharmacodynamic properties of PPIs. The genetic polymorphism of CYP2C19 is believed to be one of the major factors affecting the antibiotic resistance among* H. pylori *strains. Based on the ability to metabolize CYP2C19 substrates, three distinct phenotypes of CYP2C19 are known to exist: extensive metabolizers (EMs), intermediate metabolizers (IMs), and poor metabolizers (PMs) [13]. The distribution of CYP2C19 genetic polymorphism in the rural population of Chongqing region has not been investigated. This study represents the first systematic assessment of the antibiotic resistance profile of* H. pylori* and genetic polymorphism of CYP2C19 among the rural population in Chongqing, China. The objective is to provide a theoretical basis for the individualized therapy for* H. pylori* infection.

## 2. Materials and Methods

### 2.1. Study Subjects

The study was conducted from January 2015 to June 2015. The protocol was reviewed and approved by the Hospital Ethics Review Committee, Chongqing, China (no. (2014) 20). Subjects residing in rural areas of Chongqing region and presenting with abdominal pain, bloating, nausea, vomiting, hematemesis, or melena (*N* = 667) were recruited at 11 hospitals (Daping Hospital, Changshou District People's Hospital, Dianjiang County People's Hospital, Sanxia Central Hospital, Qianjiang Central Hospital, Qijiang County People's Hospital, Fengdu County People's Hospital, Dazu County People's Hospital, Fuling Central Hospital, Kaixian People's Hospital, and Hechuan District People's Hospital).

The inclusion criteria were as follows: (1) age: 18–75 years, male or female; (2) being positive for ^13^C/^14^C-urea breath test and* H. pylori* culture; (3) no history of treatment for* H. pylori* infection; (4) no prior administration of antibiotics, bismuth, probiotics, H2 receptor antagonists, or PPIs in the immediately preceding 4 weeks; (5) residing in rural areas of Chongqing. Exclusion criteria were as follows: (1) patients treated with gastrectomy, gastric angioplasty, or vagus nerve amputation; (2) pregnant or lactating women; (3) patients with serious liver, kidney, heart, brain, lung, endocrine, or hematopoietic disorders; (4) patients with malignant tumors; (5) patients with Zollinger-Ellison syndrome; (6) participation in other clinical studies in the last three months.

Urban patients infected with* H. pylori* (*N* = 290) were also recruited during the same period from Daping Hospital, employing the same inclusion criteria except that the subjects were required to have always been living in urban areas in Chongqing. Written informed consent was obtained from all study subjects prior to their enrolment in the study.

### 2.2. Specimen Collection

All patients were subjected to upper gastrointestinal endoscopy (UGIE). For isolation of* H. pylori*, two gastric tissue specimens were collected, one from the antrum <5 cm from the pylorus and the other from the gastric body. In addition, one gastric tissue specimen was collected from antrum for analysis of CYP2C19 gene polymorphism. All specimens were stored at −80°C until further processing.

### 2.3. Isolation of* H. pylori*

The presence of* H. pylori* in the gastric tissue samples was determined on microbiological culture. Biopsies were ground and cultured on solid selective, enriched medium (*H. pylori* agar, Becton Dickinson) and incubated microaerophilically at 37°C for 72 h. Transparent colonies were picked and subjected to gram staining to demonstrate the presence of gram-negative spiral or S shape bacteria. Isolates found to be positive for urease, catalase, and oxidase were considered as* H. pylori*.

### 2.4. Drug Susceptibility Testing (DST)

Drug susceptibility testing of* H. pylori* isolates was performed by agar dilution method. The different antibiotic solutions with desired concentrations were added to Columbia agar medium containing 5% fresh defibrinated sheep blood before being poured into plates. Fresh colonies were picked with inoculating loop and formulated into bacterial suspension with sterile saline. Two microlitres of mixture was inoculated on plates containing antibiotics and incubated microaerophilically at 37°C for 72 h. Growth in the presence of drug was regarded as being indicative of resistance, while absence of growth was considered indicative of susceptibility of the strain to that antibiotic. As per the US Clinical and Laboratory Standards Institute guidelines [14], the minimum inhibitory drug concentrations were as follows: metronidazole 8 *μ*g/mL, clarithromycin 1 *μ*g/mL, amoxicillin 2 *μ*g/mL, gentamicin 8 *μ*g/mL, levofloxacin 2 *μ*g/mL, and furazolidone 2 *μ*g/mL. NCTC11637* H. pylori *strain (43504, ATCC, USA) was used as the control strain. All the tests were conducted in Hangzhou Zhiyuan Medical Inspection Institute, China.

### 2.5. CYP2C19 Gene Polymorphism

Genomic DNA was extracted from stomach tissues using DNA extraction kit (Promega, USA) and subjected to 1% agarose gel electrophoresis. Two polymerase chain reactions (CYP2C19^*∗*^2 and CYP2C19^*∗*^3) were performed using genomic DNA extracted from gastric mucosa as a template. The primer sequences were as follows: 5′-GCAGGTATAAGTCTAGGAAATG-3′ and 5′-TAAAGTCCCGAGGGTTGTTG-3′ for CYP2C19^*∗*^2; 5′-CACCCTGTGATCCCACTTTC-3′ and 5′-CTAATGGGCTTAGAAGCCTG-3′ for CYP2C19^*∗*^3. For 25 *μ*L PCR reaction, 1.5 *μ*L of DNA sample was mixed with 2.5 *μ*L 10x Master Mix, 1.25 *μ*L 10 *μ*M of forward and reverse primers each, 2 *μ*L 10 mM dNTP, 1.5 *μ*L DNA Taq polymerase, and 15 *μ*L reverse transcriptase (RT-) PCR grade water. The PCR conditions used were as follows: initial denaturation at 94°C for 5 min, followed by 30 amplification cycles, each consisting of denaturation at 94°C for 30 s, annealing at 50°C for 30 s, extension at 72°C for 1 min, and final extension at 72°C for 5 min. PCR product was recovered by PCR recovery kit (Tiangen, China).

CYP2C19^*∗*^2 amplification products were sequenced using forward primers, while CYP2C19^*∗*^3 amplification products were sequenced using reverse primers. The sequencing results were analyzed using Chromas and Sequencing Analysis 5.2 software. Based on the mutations on CYP2C19^*∗*^2 and CYP2C19^*∗*^3, patients were categorized as belonging to one of three groups: EM (636GG, 681GG), IM (636GG, 681GA), (636AG, 681GG), and PM (636GG, 681AA), (636AG, 681AG), (636AA, 681GG).

### 2.6. Statistical Analysis

Chi-square test was used to assess potential association of antibiotic resistance profile of* H. pylori* with the following variables: gender, age, body mass index (BMI), disease type, housing area, education status, source of drinking water, smoking, and alcohol intake, as well as the CYP2C19 phenotypes. *P* < 0.05 was considered statistically significant. All statistical analyses were performed using SPSS 16.0 software.

## 3. Results

### 3.1. Antibiotic Resistance Profile of* H. pylori* in Rural and Urban Populace

214 strains were isolated from rural patients, with a mean age of 47.6 ± 10.1, 112 male and 102 female. 111 strains were isolated from urban patients, with a mean age of 46.8 ± 9.8, 70 male and 41 female.

Table 1 showed the antibiotic resistance profile of* H. pylori* isolates to six antibiotics. In rural patients, 99.53%, 20.09%, and 23.36% isolates were found to be resistant to metronidazole, clarithromycin, and levofloxacin, respectively, while none of the isolates were resistant to amoxicillin, furazolidone, or gentamicin. Resistance to metronidazole + clarithromycin/levofloxacin was found in 28.5% isolates, while that to metronidazole + clarithromycin + levofloxacin was found in 7.5% isolates. In urban patients, 82.88%, 19.82%, and 24.3% of all isolates were found to be resistant to metronidazole, clarithromycin, and levofloxacin, respectively. Resistance to metronidazole + clarithromycin/levofloxacin was found in 13.51% isolates, while that to metronidazole + clarithromycin + levofloxacin was found in 15.32% isolates. The resistance rate to metronidazole in rural patients was significantly higher than that in urban patients (*P* < 0.05).

### 3.2. Correlation of* H. pylori* Multiple Antibiotic Resistance and the Characteristics of Rural Patients

No significant correlation was observed between antibiotic resistance among* H. pylori* isolates and rural patient characteristics such as gender, body mass index (BMI), disease type, housing area, education status, source of drinking water, smoking, and alcohol intake, except age. The multiple antibiotic resistance percentage increased from 28.26% (below 45 years) to 41.80% (above 45 years) (*χ* ^2^ = 4.176, *P* < 0.05).

### 3.3. Correlation between* H. pylori* Antibiotic Resistance and CYP2C19 Genetic Polymorphism in Rural Patients

Chi-square test was used to assess differences in the antibiotic resistance profile of* H. pylori* isolated from rural patients with different CYP2C19 phenotypes. No significant correlation was observed between CYP2C19 phenotypes and resistance of* H. pylori* isolates to clarithromycin, levofloxacin, and metronidazole (Table 2).

### 3.4. Prevalence of CYP2C19 Phenotype in Rural and Urban Patients

The phenotype and frequency of CYP2C19 alleles in rural and urban patients from whom the* H. pylori* strains were isolated were compared by chi-square test. EMs, IMs, and PMs accounted for 44.39%, 45.79%, and 9.81% of positive patients in rural areas and 33.33%, 54.96%, and 11.71% in urban patients. No statistically significant difference was found in the frequency of CYP2C19 phenotypes between rural and urban patients (*χ* ^2^ = 3.706, *P* > 0.05).

## 4. Discussion

*H. pylori* is one of the most common human pathogens with a global infection rate of >50%. The prevalence of* H. pylori* infection in adult population of China is estimated to be 40–60% [2]. Treatment of* H. pylori* infection by a triple or quadruple drug regime is recommended; however, the emergence of antibiotic resistance is an important issue to be considered [15, 16]. Globally, a rising trend in the emergence of resistance to metronidazole, clarithromycin, and levofloxacin among* H. pylori* isolates has been acknowledged [17]. With the widespread use of antibiotics for treatment of* H. pylori* infection, the phenomenon of antibiotic resistance is expected to gradually increase. Indeed, 60% to 70% isolates of* H. pylori* are reportedly resistant to metronidazole [18].

A prospective study conducted in Beijing reported 66.8% and 63.4% prevalence of resistance to metronidazole among* H. pylori* isolates in years 2009-2010 and 2013-2014, respectively [19]. In the present study, 99.6%* H. pylori* isolates from rural patients were found to be resistant to metronidazole, which was significantly higher than that reported from other parts of China and abroad [20, 21]. This is likely related to the misuse of antibiotics and the generally lax control over the sale of antibiotics. However, further epidemiological investigations are required to identify the specific reasons.

In view of the results of our study, metronidazole should not be used for treatment of* H. pylori* infection, particularly in the rural areas of Chongqing. Clarithromycin is one of the main components of the standard triple therapy for treatment of* H. pylori* infection. The increased resistance to clarithromycin is liable to directly impact on the eradication rates of* H. pylori* [22]. In the present study, 20.1% of* H. pylori* isolates were resistant to clarithromycin, which is in line with that reported in a recent report [23].

According to the Maastricht IV/Florence Consensus Report [24], if >15–20% isolates of* H. pylori* develop resistance to clarithromycin, nonbismuth quadruple therapy should be avoided. Therefore, in rural areas of Chongqing, clarithromycin should only be prescribed based on the results of drug susceptibility testing. The resistance rate to levofloxacin was found to be 22.2%, which is consistent with the estimated national prevalence [25]. None of the* H. pylori* isolates were found to be resistant to amoxicillin, furazolidone, and gentamicin. These three drugs may be used in the absence of data on drug susceptibility.

Antibiotic resistance among* H. pylori* strains manifests considerable geographical variations [26]. In the present study, we not only investigated the resistance rate in patients from rural areas in Chongqing, but also demonstrated a lack of correlation between the* H. pylori* antibiotic resistance and gender, body mass index (BMI), disease type, housing area, education status, source of drinking water, smoking, and/or alcohol intake, except age. Resistance to multiple antibiotics is more likely in patients over the age of 45 than those less than 45 years (*P* < 0.05). Guo et al. [27] demonstrated that the antibiotic resistance rate of* H. pylori* increased with age, which might be due to the antibiotic accumulation. Our findings suggest the relevance of the notion of individualized medicine in the clinical treatment of* H. pylori*, especially in the rural population, where a 28.5% prevalence of resistance to double drugs (metronidazole + clarithromycin/levofloxacin) and 7.5% prevalence to triple drug therapy (metronidazole + clarithromycin + levofloxacin) were observed.

Several reasons are attributed to bacterial resistance to antibiotics, such as frequent consumption of antibiotics, so that the resistant bacteria survive the harsh environment and could then spread the resistance genes, an intrinsic property or an occurring mutation in the chromosomal genes, such as CYP2C19. CYP2C19 is one of the most important drug-metabolizing enzymes belonging to CYP450 family and is mainly found in microsomes of hepatocytes. On the basis of their ability to metabolize CYP2C19 substrates, individuals can be classified as extensive metabolizers (EMs), intermediate metabolizers (IMs), or poor metabolizers (PMs) [13]. Genetic polymorphism (mainly CYP2C19^*∗*^2, CYP2C19^*∗*^3, and CYP2C19^*∗*^17) exists for CYP2C19 expression [28]. Two common mutant alleles of the CYP2C19 gene among the Asian population are CYP2C19^*∗*^2 and CYP2C19^*∗*^3 [29].

Prevalence of CYP2C19 gene polymorphism varies in different ethnic groups [30]. EMs are known to account for a vast majority of Europeans, while in the Asian population, PMs and EMs constitute a significant proportion. This phenomenon was believed to lead to marked individual differences in drug efficacy [31]. CYP2C19 mutations could influence the stomach pH value and thus affect the activity and stability of antibiotics, such as amoxicillin and clarithromycin [32]. Bacteria are not destroyed by these antibiotics which are not fully active in an acid environment but are exposed to subinhibitory concentrations of drugs for many hours. This might lead to the microevolution of bacteria and the appearance of resistant strains.

The present study showed that the distribution of CYP2C19 gene polymorphism in the rural population in Chongqing is in agreement with that of the Asian population [33]. No significant difference in CYP2C19 gene polymorphism was found between urban and rural populations of Chongqing. Moreover, genetic polymorphism of CYP2C19 was not found to influence antibiotic resistance profile of* H. pylori* isolated from rural population. This is the first study to investigate antibiotic resistance of* H. pylori* and distribution of CYP2C19 gene polymorphism in rural population in Chongqing.

In summary, due to the high prevalence of* H. pylori* strains resistant to metronidazole, clarithromycin, and levofloxacin in rural patients, the choice of therapy in this area should be based on local susceptibility patterns. Metronidazole should not be used for the treatment of* H. pylori* infection in the rural areas of Chongqing, China. Amoxicillin, gentamicin, and furazolidone are recommended as the first-line empiric therapy regimens.

## Figures and Tables

**Table 1:** Results of drug susceptibility tests of *H. pylori* among the rural and urban population in Chongqing.

Antibiotics	Antibiotic resistant cases (*n*) and rates (%)		*χ* ^2^	*P* value
	Rural	Urban		
Metronidazole	213 (99.53)	92 (82.88)	35.084	0.000
Clarithromycin	43 (20.09)	22 (19.82)	0.003	0.953
Levofloxacin	50 (23.36)	27 (24.32)	0.037	0.847
Amoxicillin	0	0		
Furazolidone	0	0		
Gentamicin	0	0		

**Table 2:** Correlation between *H. pylori* antibiotic resistance and CYP2C19 genetic polymorphism in rural patients.

CYP2C19 phenotype	Antibiotic resistant cases (*n*) and rates (%)		
	Clarithromycin	Levofloxacin	Metronidazole
EMs	21 (22.11)	17 (17.89)	95 (100)
IMs	16 (16.33)	20 (20.41)	97 (98.98)
PMs	6 (28.57)	3 (14.29)	21 (100)
*P* value	0.360	0.780	NS

EMs, extensive metabolizers; IMs, intermediate metabolizers; PMs, poor metabolizers.

NS: not significant by chi-square test.
